# Pericytes in Glioblastoma: Hidden Regulators of Tumor Vasculature and Therapy Resistance

**DOI:** 10.3390/cancers17010015

**Published:** 2024-12-24

**Authors:** Irene Salazar-Saura, María Pinilla-Sala, Javier Megías, Lara Navarro, Esther Roselló-Sastre, Teresa San-Miguel

**Affiliations:** 1 Pathology Service, Consorcio Hospital General Universitario de Valencia, 46014 Valencia, Spain; salazar_ire@gva.es (I.S.-S.); lara.navarro@uv.es (L.N.); rosello_est@gva.es (E.R.-S.); 2 Research Group on Tumors of the Central Nervous System, Pathology Department, University of Valencia, 46010 Valencia, Spain; merypinisala@gmail.com; 3 INCLIVA Foundation, 46010 Valencia, Spain

**Keywords:** pericyte, intussusception, vascular mimicry, DDR, PDGFR-β, NG2, VEGF

## Abstract

Glioblastoma IDH wild type (GB), the most common malignant primary brain tumor, is characterized by rapid proliferation, extensive infiltration into surrounding brain tissue, and significant resistance to current therapies. Median survival is only 15 months despite extensive clinical efforts. The tumor microenvironment (TME) in GB is highly specialized, supporting the tumor’s aggressive behavior and its ability to evade conventional treatments. One critical component is the aberrant vascular network that complicates the delivery of chemotherapy across the blood–brain barrier. Antiangiogenic therapies emerged as a promising option but have shown limited efficacy in extending the survival of these patients. Comprehension of the complex vascular network of GB may be a key to overcoming the limitations of current therapies. Pericytes are gaining recognition within the context of the TME. These mural cells are essential for vascular integrity and may contribute to tumor progression and therapeutic resistance. Although their role has been evidenced in other tumors, they remain underexplored in GB. Pericytes are known to respond to tumor hypoxia and interact with vascular endothelia, influencing responses to DNA damage and antiangiogenic treatments. They actively regulate not only angiogenesis but also the different vasculogenic strategies for tumor neovascularization. Additionally, they affect leukocyte trafficking and tumor-associated macrophages. This review aims to integrate the various functions controlled by pericytes to favor deeper investigation into their actionable potential. Pericytes may represent a promising target for novel therapeutic strategies in order to improve patient outcomes.

## Simple Summary

Glioblastoma is the most common and aggressive type of primary brain cancer, known for its rapid growth, invasion into surrounding brain tissue, and strong resistance to current therapies. Despite intensive treatment efforts, patients have a median survival of just 15 months. One major treatment challenge is the tumor’s complex vascular network which causes the limited success of antiangiogenic therapies in extending patients’ survival. Pericytes are cells essential for maintaining the integrity of this vascular network. Additional to their structural functions, they play diverse roles that support tumor progression and treatment resistance. This review aims to understand the multifaced functions of pericytes in the glioblastoma microenvironment; the comprehension of those roles may guide new therapeutic strategies to improve outcomes for patients affected with this devastating disease.

## 1. Introduction

Glioblastoma (GB) is the most aggressive and lethal form of primary brain tumor, classified as a WHO grade 4 adult-type diffuse glioma. It is characterized by rapid proliferation, extensive infiltration into surrounding brain tissue, and remarkable resistance to current therapies. The median survival is just 15 months. Despite significant efforts, only modest improvements have been achieved with intensive multimodal treatment, which includes radical tumor resection followed by a STUPP combined chemotherapy–radiotherapy protocol [1]. The 2016 WHO classification introduced a major shift in glioma taxonomy by incorporating molecular markers, distinguishing glioblastoma IDH-wild-type (IDH-wt) from IDH-mutant astrocytomas. In the 2021 update, this framework was refined, separating high-grade gliomas (HGGs) in adults from those occurring in children, given their distinct molecular and clinical features. In adults, GB is predominantly IDH-wt, with hallmark alterations including EGFR amplification, TERT promoter mutations, and chromosome 10 q loss. Conversely, pediatric HGGs, such as diffuse midline gliomas (DMGs), often harbor unique mutations, including H3 K27M, which are absent in adult counterparts. These differences contribute to varying clinical presentations, responses to therapy, and survival outcomes, with pediatric gliomas generally exhibiting even worse prognosis [1].

The tumor microenvironment (TME) in GB is highly specialized, supporting both the aggressive nature of the tumor and its ability to evade conventional treatments. GB is one of the most vascularized tumors, and its aberrant vascular network is a key component of the TME [2]. The behavior of this complex vascular network and the cellular components that regulate it remains incompletely understood, particularly in the immature and disorganized vasculature of pediatric gliomas. These knowledge gaps represent a major limitation in current therapeutic approaches. Trespassing the blood–brain barrier (BBB) in those intricate and abnormal vascular networks is challenging for chemotherapy. Antiangiogenic therapies initially emerged as a promising therapeutic option for GB patients; however, their efficacy in improving overall survival has been limited [3].

Pericytes are gaining recognition as pivotal players in the TME of GB. These mural cells are essential in maintaining vascular integrity and may also contribute to tumor progression and therapeutic resistance [4]. Although their roles have been studied in other tumors, their specific contributions to adult and pediatric gliomas remain underexplored; their characteristic aberrant vascular networks may be influenced by pericyte-mediated resistance mechanisms. Given the role of pericytes in angiogenesis, immune regulation, and therapeutic resistance, this review focuses on their involvement in adult IDH-wild-type glioblastoma. It examines their biological properties, hypoxia-driven activation, and role in tumor pathophysiology. A deeper understanding of these processes could uncover novel therapeutic strategies to improve patient outcomes.

### 1.1. Pericytes Are Mural Cells Sensible to Hypoxia

**Figure 1:**
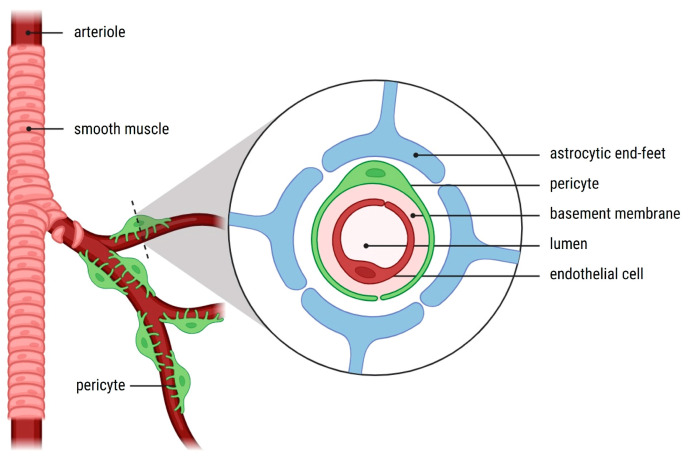
Pericyte interactions in the neurovascular unit. Pericytes are strategically positioned in capillaries, where they play a key role in regulating the blood–brain barrier and vascular homeostasis. In this image, a pericyte is shown enveloping the arteriole and making direct contact with endothelial cells through the vascular basement membrane. The astrocytic end-feet, located above the pericytes, contribute to reinforcing the barrier and modulating neurovascular interactions.

Pericytes are contractile cells that reside on the abluminal surface of capillaries and microvessels (Figure 1). They regulate vascular stability and permeability, maintain the BBB and play a role in the formation of the glial scar in pathological conditions. They exhibit stem cell-like properties as well [5]. Brain vessels are rich in these mural cells, and pericytes can be recruited to hypoxic regions thanks to their migratory properties [5,6]. In GB, pericytes support vessel formation with an atypical vascular pattern. The existence of pericytes that are abnormally thickened and extensively present is a characteristic feature of microvessels in high-grade gliomas; in fact, some cases show a microvasculature net composed only of pericytes, even without endothelial cells (ECs), and they show a high proliferative index [6,7,8]. To better study and identify pericytes within the TME, specific proteins expressed by these cells can serve as immunohistochemical markers:

- PDGFRβ (platelet-derived growth factor receptor beta) [9].
- NG2 (neuroglial 2 antigen): a proteoglycan widely used for pericyte identification in various tissues, including the brain [9].
- αSMA (smooth muscle actin alfa): it identifies contractile pericytes in tissues [10].
- Some CD markers, like CD13 and CD146 (MCAM) [11] and CD54 (ICAM-1).
- RGS5 (regulator of G-protein signaling 5): associated with GB pericytes and pathological angiogenesis [12].

Pericytes originate from mesenchymal progenitors. Microenvironmental signaling plays a key role in specific differentiation, dependent on PDGF-β signaling. Thus, PDGF-B is secreted by ECs in conditions of hypoperfusion and promotes PDGFR-β expression in pericytes and their migration towards newly formed immature microvessels in the BBB. In the vessel maturation process, VEGF (vascular endothelial growth factor) acts as a necessary factor. VEGF-A165 increases through pericyte PDGFR-β signaling, dependent on PI3K (phosphatidylinositol 3-kinase) activation [13]. VEGF-A165 binds to heparan sulfate proteoglycans on the cell surface and facilitates both local EC survival and vessel stabilization, but not EC migration [14].

In pathological conditions, pericyte origin remains controversial. One hypothesis suggests that they may originate from glioma stem cells (GSCs). GB exhibits a cellular hierarchy dominated by glioma stem cells (GSCs). These cells are characterized by their self-renewal capacity and ability to differentiate into multiple lineages. GSCs can recapitulate the original tumor phenotype and adapt to hostile microenvironments. Moreover, they are highly resistant to both radio and chemotherapeutic agents [15,16]. GSCs can be identified by the expression of specific markers: CD133 is associated with GB capacity for self-renewal and therapy resistance, and OCT4 and SOX2 are key regulators of stemness that support plasticity and adaptability to the microenvironment.

**Figure 2:**
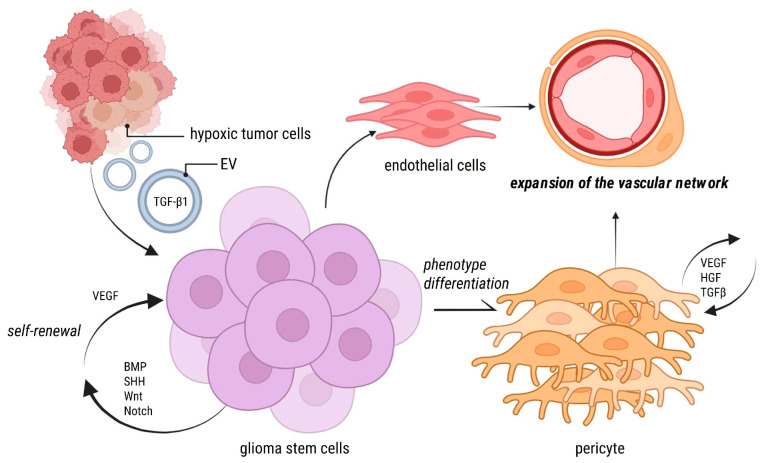
GSCs as potential reservoirs of vascular cells. Glioblastoma stem cells (GSCs) cooperate to ensure tumor vascularization through multiple mechanisms. Autocrine VEGF signaling promotes GSC self-renewal and proliferation, but also their differentiation into pericyte-like cells. Pericyte signaling contributes to endothelial maturation and vessel stabilization. Hypoxia within the tumor microenvironment enhances the release of small extracellular vesicles (EVs) containing factors such as TGF-β1, which modulate GSC behavior and further amplify vascular support. This is an example of the coordinated systems used by GSCs and pericytes to maintain glioblastoma growth and angiogenesis. Abbreviations: EV, extracellular vesicles; GSCs, glioma stem cells; GB, glioblastoma IDH wild type.

It has been proposed that these GSCs reside in at least two distinct niches within a tumor: adjacent to capillaries, interacting with ECs and releasing VEGF to stimulate angiogenesis; and in the hypoxic niche, with a crucial regulatory role in inducing the expression of self-renewal genes and regulating cellular differentiation [16]. In both cases, the location near ECs and the plasticity of GSCs support how GSCs can acquire a pericyte phenotype (Figure 2) [15,17,18]. A second hypothesis postulates an endogenous origin, based on other works where more than half of all pericytes within the tumor bulk are endogenous pericytes [17]. Interestingly, both hypotheses share a fundamental point: hypoxia is a catalyst for the activation of pericytes and triggers a series of downstream signals. Beyond their response to hypoxia, pericytes establish critical interactions with endothelial cells, modulating vascular stability, angiogenesis, and GB progression. Additionally, a recent study demonstrated that hypoxia-inducible factor-1α (HIF-1α) enhances the expression of platelet-derived growth factor β (PDGF-β) in endothelial cells, promoting communication with pericytes. This signaling pathway is pivotal in hypoxic conditions, driving angiogenesis and sustaining the abnormal vascular microenvironment in glioblastoma [19].

### 1.2. Pericytes Interact with Endothelial Cells

The abnormal vascular network found in GB is driven by hypoxia and tumor-derived signals, supplying nutrients and accelerating tumor progression. Although not well understood, there is an intrinsic communication between ECs and pericytes which modulate neovascularization processes. The regulatory role of pericytes in vascular stability and permeability in oncological contexts has gained attention [19,20,21]. Through interactions with ECs, pericytes overexpress the transmembrane receptor endosialin (CD248 or TEM1), which takes part in maintaining the microvasculature net exclusively in malignant solid tumors, including high-grade gliomas [17,22]. Other signaling pathways, such as the CXCL12-CXCR4 axis [23] and the EphrinB2–EphB4 system, mediate pericyte–EC communication [6,24,25].

Pericytes secrete proangiogenic factors such as VEGF (vascular endothelial growth factor), hepatocyte growth factor (HGF), prostaglandins, and TGF-β1. In fact, pericytes produce large quantities of TGF-β, which enhances their own expression of VEGFR-1 and VEGF. This results in the upregulation of angiopoietin-1 (Ang-1) signaling and its receptor Tie-2, facilitating pericyte–EC communication via a paracrine loop and contributing to endothelial maturation and vessel stabilization [21,26].

Neuron-glial antigen 2 (NG2), also known as chondroitin sulfate proteoglycan 4 (CSPG4), is a membrane component highly expressed in angiogenic pericytes and GB cells [27]. In a melanoma model, it has been shown that the ablation of NG2 in pericytes increases vascular leakage and intra-tumoral hypoxia, weakens zonula occludens-1 (ZO-1) junctions, increases endothelial monolayer permeability, and impairs endothelial integrin β1 activation [28]. In GB, NG2 isoforms were identified in proliferative perivascular pericytes, but not in non-neoplastic cells. Those pericytes are implicated in early angiogenesis, initiating processes such as sprouting angiogenesis and vasculogenic mimicry [29,30]. NG2 pericyte overexpression induces integrin β1-mediated mechanisms to maintain the abnormal but functional vascular network in GB during tumor progression [27,31].

Pericytes have recently been classified into two subtypes. Type-1 pericytes do not contribute directly to new vessel formation, but exposure to TGF-β can induce a tumor-associated fibroblast phenotype in these cells, producing extracellular matrix components, which in turn promotes angiogenesis indirectly [8,14]. Type-2 pericytes are capable of inducing angiogenesis when co-cultured with ECs, or when injected in vivo in GB. Only these type-2 pericytes are recruited to sites of tumor angiogenesis and can be recognized by nestin expression [14].

## 2. The Pericyte as a Key Mediator in Therapeutic Failure

Small niches of GB cells that survive after therapy have been described in perivascular aggregates, often expressing stem-like markers associated with self-renewal and therapy resistance such as CD133, OCT4, SOX2. These findings led to further investigations into whether stem-like cells in that location might adopt endothelial or pericytic characteristics, thereby contributing to tumor relapse [16,18]. These tumor cells displaying endothelial and pericyte phenotypes demonstrated the ability to initiate tumor growth in xenotransplantation models [16]. These observations suggest that GSCs residing in perivascular niches could support tumor vasculature through different mechanisms, providing a reservoir of multipotent vascular progenitors that facilitate tumor persistence and progression (Figure 2) [32] as we well discuss afterwards.

### 2.1. DNA Damage Repair Response (DDR) and Pericytes

Pericytes contribute to therapeutic resistance in GB by modulating the DNA damage repair (DDR) response. A key pathway involved in this process is the CCL5-CCR5 signaling axis, which protects tumor cells from therapy-induced DNA damage. Zhang et al. explored the role of pericytes in GB resistance to TMZ. They found that pericytes secrete CCL5 (C-C motif chemokine ligand 5), a chemokine that modulates immune responses and promotes cell migration. CCL5 binds to its receptor CCR5 (C-C chemokine receptor type 5) which is expressed on GB cells. This interaction, in response to TMZ, triggers a signaling cascade that activates the DDR response. Specifically, it induces AKT phosphorylation and activates DNA-PKcs (DNA-dependent protein kinase catalytic subunit), an enzyme in the non-homologous end joining (NHEJ) pathway [33,34]. AKT phosphorylation enhances the cellular response to DNA damage, protecting GB cells from TMZ-induced apoptosis. DNA-PKcs repairs DNA double-strand breaks, ensuring that DNA lesions induced by TMZ are efficiently repaired. This process reduces the cytotoxic impact of the treatment. This pericyte–tumor cell communication, mediated by the CCL5-CCR5 pathway, is a good example of how a microenvironment supports GB cells during therapeutic stress. Similar mechanisms have been observed in other tumor types, such as lung cancer and melanoma [35]. These findings underscore the dual role of pericytes, not only as vascular support cells but also as active mediators of therapeutic resistance in glioblastoma.

### 2.2. Resistance to Anti-VEGF Strategies

Anti-VEGF therapies, designed to target tumor angiogenesis, face significant challenges due to compensatory mechanisms within the tumor microenvironment. Pericytes play a critical role in these resistance processes by stabilizing vasculature and promoting alternative angiogenic pathways. It has been observed that, although anti-VEGF therapies in GB initially reduce endothelial cell (EC) density, this reduction is followed by increased pericyte vessel coverage. This stabilization promotes endothelial survival and vascular function [36]. An accumulation of PDGFR-β+ stromal cells is found in these *foci*, inducing angiogenesis [10].

GSCs have shown significant plasticity in hypoxic conditions, such as the situation generated by anti-VEGF therapy. Under those circumstances, GSCs release extracellular signals helping themselves to transition into a pericyte-like phenotype [17,18]. This signaling is, in part, mediated by glioma-derived extracellular vesicles (sEVs) [15,37,38,39,40] which efficiently transport TGF-β1 to GSCs (Figure 2). TGF-β activation maintains angiogenesis and repairs blood vessel leakage, counteracting the effects of VEGF inhibition. These interactions accelerate tumor infiltrative growth, complicating surgical resection and increasing recurrence risks [15,41,42].

In addition, pericytes activate alternative pathways involved in resistance to anti-VEGF and anti-PDGF therapies, such as the EphrinB2–EphB4 axis [6]. While VEGF remains a primary target in antiangiogenic strategies, other angiogenic pathways, such as PDGF and FGF, can be simultaneously targeted using multi-tyrosine kinase inhibitors (e.g., sorafenib, sunitinib, and pazopanib). However, these multi-target therapies may activate distinct resistance mechanisms mediated by pericytes, including pericyte recruitment and vessel stabilization under VEGF blockade [43,44]. It is worth mentioning that the clinical efficacy and safety profiles of antiangiogenic therapies differ between adults and pediatric patients with high-grade gliomas (HGGs). Bevacizumab has shown limited efficacy in pediatric HGGs compared to in adult GB, with lower response rates and minimal impact on survival; although antiangiogenic therapies are generally well tolerated in children, adverse events (AEs) remain a significant concern. In aggressive pediatric gliomas, the vascular network frequently exhibits immature and disorganized features, resulting in increased permeability. These structural abnormalities may limit the efficacy of antiangiogenic strategies [43,44]. Unfortunately, the specific role of pericytes in pediatric gliomas’ aberrant vasculature remains poorly understood, as most studies have focused on adult gliomas. These observations underscore the complexity of antiangiogenic resistance mechanisms in GB and pediatric gliomas and highlight the need for further research to optimize therapeutic strategies across different patient populations.

## 3. Pericytes Are Active Agents in GB Neovascularization

A surprising mechanistic finding about another way by which pericytes regulate angiogenesis both in normal and pathological situations is their ability to emit tunneling nanotubes (TNTs). TNTs are tubular structures firstly described in 2004 that are involved in communication between distant cells. Errede et al. demonstrated that pericytes are the primary source of TNTs during vessel growth and branching in fetal brain development and in GB. Pericytes play a crucial role in cellular recognition, connection, and communication through their TNT-mediated “vascular wiring” in both normal and pathological angiogenesis [45]. Climent et al. hypothesize that pericyte TNTs transfer microRNAs that are able to modulate angiogenesis and vessel stabilization [46].

GT198 (*PSMC3IP* or Hop2), an oncoprotein encoded by a DNA repair gene, is overexpressed in tumor vessels. Zhang et al. showed that the expression of GT198 by some pericytes in diverse tumors, including GB, confers a malignant behavior [47]. These “malignant pericytes” not only stabilize new vessels but also take part in their growth, as is supported by the effect achieved by vaccination against GT198 that affected pericyte GT198+ and reduced tumor angiogenesis [48]. This is some evidence that reinforces the vasculogenic role of malignant pericytes.

It is worth including in this review a brief description of neovascularization models in GB to understand the wide range of effects of pericyte functions described in all of them. Briefly, pericyte participation has been described both in angiogenic mechanisms, in vascular mimicry, and in other vasculogenic models.

### 3.1. Role of Pericytes in Glioblastoma Angiogenic Mechanisms

**Figure 3:**
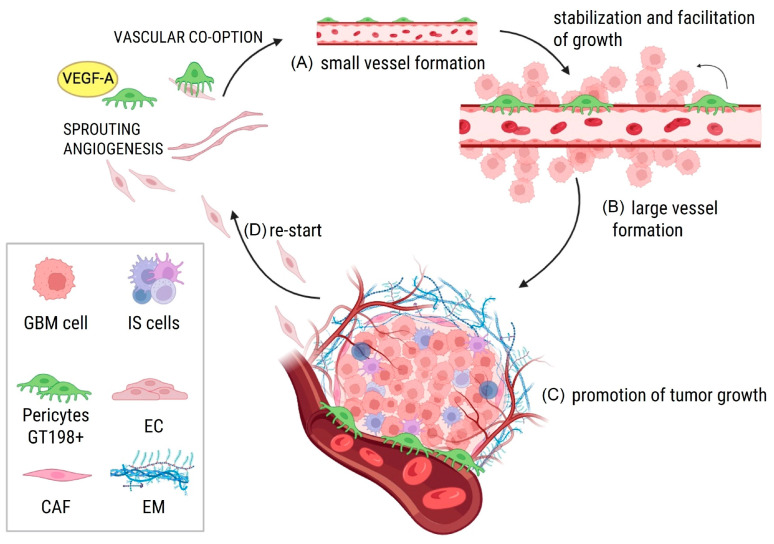
Hypothetical model integrating various theories of angiogenesis involving pericytes in GB. (**A**) Sprouting endothelial cells, supported by pericytes, initiate the formation of new blood vessels by co-option; (**B**) pericytes in the tumor microenvironment stabilize these nascent vessels and, as progenitor-like cells, contribute to their growth and maturation into functional, larger vessels; (**C**) the expanding vascular network supplies nutrients and oxygen, supporting tumor growth and progression; (**D**) pericytes actively participate in successive angiogenic cycles, remodeling the vasculature to adapt to tumor demands. Altogether, this promotes glioblastoma invasion. Interactions with cancer-associated fibroblasts (CAFs), extracellular matrix (ECM) components, and immune cells further modulate these processes. Abbreviations: GB, glioblastoma; ECs, endothelial cells; CAFs, cancer-associated fibroblasts; ECM, extracellular matrix.

Angiogenesis is the formation of new blood vessels from pre-existing vessels. There are three principal mechanisms: sprouting angiogenesis, vascular co-option, and intussusceptive angiogenesis (Figure 3). In *sprouting angiogenesis*, pericytes in the surrounding area produce elevated levels of VEGF that stimulate the sprouting of ECs from pre-existing capillaries, directing them toward the pericytes [21]. *Vascular co-option* involves the utilization of pre-existing vessels by malignant pericytes, which capture and enclose endothelial cells, forming small blood vessels [17]. Pericytes, positioned on the abluminal side of blood vessels, facilitate these processes by modulating vessel morphology through cytoskeletal reorganization mediated by proteins such as Cdc42 [12,49,50]. Caspani et al. were the first to show, using two-photon excitation microscopy and live confocal imaging, that GB cells form actin-based extensions, called flectopodia, alter pericyte contractility. Through them, cells can transfer Cdc42, leading to reorganizations of blood vessel morphology. They demonstrated that inhibiting Cdc42 prevents both vascular co-option and vascular mimicry (described below) in GB [51]. All these findings support that GB cells take part in co-opting angiogenesis with the assistance of pericytes to proliferate and progress. *Intussusceptive angiogenesis*, also called *intussusception,* also depends on pre-existing microvasculature but does not require endothelial cell proliferation. The mechanism is based in the insertion of connective tissue columns, called tissue pillars, into the lumen and the subsequent growth of these pillars, resulting in partitioning of the vessel lumen. The formation of the tissue pillars is preceded by the emergence of vessel wall folds, but the mechanisms behind these invaginations have not yet been clarified. Anyway, the consequence of the partitioning of the vessels is a rapid increase in the density of the capillary network. This division and remodeling is facilitated by the abundance of proliferating malignant pericytes and is due to the lack of quiescent normal pericytes maintaining vessel stability [31]. This process is more energy- and time-efficient compared to sprouting angiogenesis, as it does not require the proliferation and migration of endothelial cells.

These angiogenic mechanisms depend on pre-existing vessels with endothelial cells. However, GB can also use alternative strategy networks without the involvement of ECs or pre-existing vessels to maintain its vascular network. In these processes, pericytes are essential for stabilizing the abnormal structures, further promoting tumor growth.

### 3.2. Role of Pericytes in Glioblastoma Vascular Mimicry

The de novo formation of vascular structures by tumor cells without the involvement of endothelial cells is described as *vascular mimicry* (VM). This is a process in which GB cells adopt a vascular phenotype. In VM, tumor cells form tubular structures that mimic blood vessels, acting as fluid-conducting channels while expressing endothelial markers [52]. Vascular mimicry represents the amazing ability of tumor cells to create pseudovascular networks that support their rapid growth and invasiveness. Unlike normal angiogenesis, VM allows tumors to form these vascular-like networks independently of endothelial cells. Pericytes also play a supportive role in stabilizing these abnormal structures [53]. Ultrastructural studies of GB show that the presence of a continuous ring of tumor cells adhered to the luminal side of the vascular wall in many cases, which appears to replace or mimic endothelial function, a clear sign of VM [54]. This angiogenesis driven by GB cells contributes to rapid tumor growth by facilitating vascular invasion, perivascular migration, and infiltration of malignant cells into surrounding tissue [55].

### 3.3. Role of Pericytes in Glioblastoma Vasculogenesis

Vasculogenesis is the term for referring to the formation of blood vessels, from progenitor or stem cells. As aforementioned, GSCs can acquire a pericyte phenotype, or they can differentiate into malignant pericytes, contributing to the vascular structure of GB [15,17,18]. Endothelial cell Notch signaling has been well characterized, while pericyte Notch signaling is less understood. It has been described that activation of the Notch-1 intracellular domain (NICD) induces the expression of pericytic markers in GSCs, like NG2, PDGFR-β, and α-SMA, while not inducing endothelial differentiation (CD31- and vWF-negative) [56]. The primary Notch ligand that stimulates angiogenesis is Delta-like ligand 4 (Dll4). Dll4 inhibition generates hypervascular tumors, but tumor growth is limited due to the lack of functionality of these branched vessels; this phenomenon is called the “delta paradox”. These non-functional vessels show a reduced pericyte coverage [57].

Other stem cells implicated in GB vasculogenesis are myeloid stem cells, derived from bone marrow. They may differentiate into pericyte progenitors and contribute to neoangiogenesis [58]. This process is driven by CXCL12 (C-X-C motif chemokine ligand 12), a chemokine regulated by HIF-1α (hypoxia-inducible factor 1-alpha). Under hypoxic conditions, common in the glioblastoma microenvironment, HIF-1α is stabilized and induces the expression of CXCL12. This chemokine mobilizes hematopoietic-derived cells, including a small fraction of pericyte progenitors, and recruits them to the tumor, where they contribute to vascular remodeling. It has been described that this mechanism depends on the presence of MMP9 (matrix metalloproteinase 9), an enzyme secreted by tumor cells and tumor-associated stromal cells, which plays a critical role in degrading the extracellular matrix. MMP9 facilitates the release of VEGF (vascular endothelial growth factor) that is sequestered in the tumor extracellular environment. The liberation of VEGF promotes angiogenesis and the incorporation of vascular-modulating cells into the growing tumor network [59].

Through these diverse neovascularization mechanisms, pericytes cyclically contribute to tumor expansion by promoting the formation of new vessels that support the tumor progression in GB.

## 4. Influence of Pericytes on the Immune System

Pericytes, in addition to their role in vascular stability and angiogenesis, also function as regulators of the immune response. Their strategic location in the perivascular niche and their ability to interact with both innate and adaptive immune cells make them interesting mediators of immune modulation. In GB, evidence supports their immunosuppressive role in facilitating tumor growth and progression.

### 4.1. Pericytes’ Role in Leukocyte Trafficking

The perivascular location of pericytes within the Virchow–Robin space, where they are in contact with cerebrospinal fluid and astrocytic end-feet, positions them ideally to control various aspects of the immune response in the central nervous system (CNS) [12]. The interaction between mural cells and immune cells has not been widely studied but it is well established that pericytes play a supportive role in the CNS immune response. This includes the expression and secretion of immunoactive molecules, the regulation of leukocyte trafficking to inflammation sites, and an early involvement in neutrophil migration. Collectively, these functions help to maintain the brain–immune interface function [60,61].

Pericytes express ICAM-1 (intercellular adhesion molecule 1), an essential protein for leukocyte adhesion and transmigration, which facilitates activation of the neuroimmune response and enhances CNS defense. Different studies indicate that PDGF-β and NG2 are involved in modulating ICAM-1 expression. These proangiogenic molecules are significantly upregulated in GB vasculature compared to in normal brain tissue supporting a role for pericytes in the immune environment of GB [27,62]. In a mouse model deficient in pericytes (*PDGFR-β* ^−/−^), ICAM-1 expression was significantly elevated in cerebral vessels, resulting in increased leukocyte infiltration into the brain [62]. Similarly, the downregulation of *NG2* induced the phosphorylation of ERK1/2s (extracellular signal-regulated kinases 1 and 2). ERK1/2s are key components of the mitogen-activated protein kinase (MAPK) signaling pathway, which regulates a wide range of cellular processes, including gene expression, proliferation, and immune response. This phosphorylation of ERK1/2s resulted in the upregulation of ICAM-1 on pericytes, promoting leukocyte adhesion to these cells and contributing to a higher immune cell presence in the vasculature [27]. These findings support that tumor-associated pericytes modulate immune cell activity within cerebral vessels and limit leukocyte infiltration into the brain parenchyma.

In addition to their role in innate immunity, pericytes also contribute to adaptive immune functions in the CNS. They express major histocompatibility complex (MHC) class I and II molecules enabling them to function as antigen-presenting cells. There is increasing evidence that GSC-derived pericytes are able to inhibit T-cell activation [63]. Perivascular pericyte cultures—characterized by CD248+, CD90+, and PDGFR-β+ markers—exhibit the ability to suppress both allogeneic T-cell responses such as T-cell activation against non-self-antigens and mitogen-activated T-cell responses, which are responses triggered by polyclonal activation, similarly to the immunosuppressive behavior observed in GSCs. The same study explored whether immature brain pericytes use similar immunosuppressive mechanisms to those of GSCs to mediate immunosuppression: they were confirmed to have in common the expression of immunosuppressive factors such as TGF-β (transforming growth factor beta) and HGF (hepatocyte growth factor), which inhibit T-cell proliferation and activation [41]. Additionally, prostaglandin E synthase promoted the production of prostaglandin E2 (PGE2), a lipid mediator with potent immunosuppressive effects on T-cells. Finally, HLA g (human leukocyte antigen G), a non-classical MHC class I molecule, inhibited T-cell and natural killer (NK) cell responses [64]. To sum up, these works suggest that pericytes can limit the adaptive immune responses against GB by presenting antigens and secreting immunosuppressive molecules.

### 4.2. Pericytes Are Close Allies of Tumor-Associated Macrophages

Pericytes exhibit dynamic immune properties within the GB microenvironment, acting as modulators of both angiogenesis and immune responses. Thess cells express innate immune system receptors, such as Toll-like receptor 4 (TKR4), whose activation triggers the expression of several macrophage-associated markers. These include CD11b (integrin αM), CD163, Fc receptors, scavenger receptors, and complement receptor CR3. These receptors facilitate phagocytosis, supporting the concept that pericytes perform immune-like functions within the CNS [60,65]. In vitro studies have shown that brain-derived pericytes posses phagocytic capacity and secrete inflammatory cytokines such as TNF-α and IL-6 when stimulated with lipopolysaccharides or cytokines [60,65].

The interaction between pericytes and tumor-associated macrophages (TAMs) is an active area of research. Pericytes can alter their immune activation state depending on the context of disease. Under acute neuroinflammation, pericytes stimulated by pro-inflammatory cytokines, such as TNF-α, release additional pro-inflammatory factors that upregulate iNOS and IL-1β expression in macrophages. This activation can induce a phagocytic state in microglia that contributes to tissue remodeling [66]. While this response may initially be protective, the resulting inflammatory environment can also facilitate glioma cell infiltration. In contrast, GB is characterized by a highly immunosuppressive microenvironment with significant TAM infiltration. Within this chronic inflammatory microenvironment, pericytes express immunosuppressive molecules such as IL-10 and TGF-β, which inhibit microglial activation and reduce their phagocytic capacity. This results in an “inactivated” microglial state, allowing the tumor to evade immune surveillance and facilitating its progression [67].

**Figure 4:**
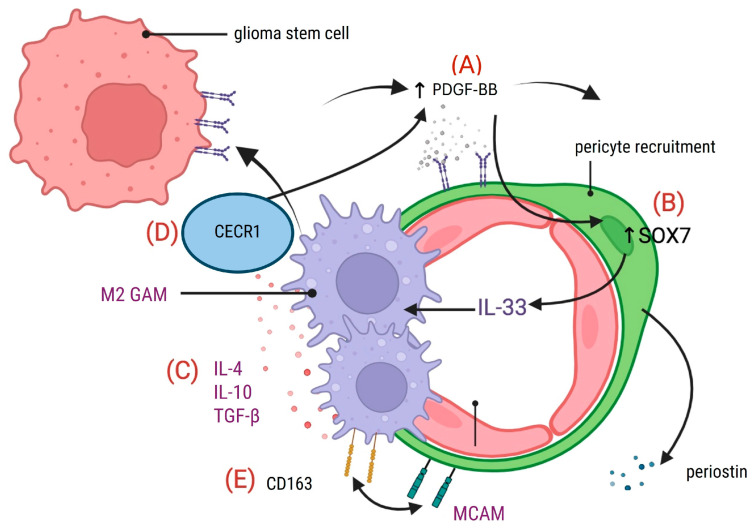
Reciprocal interactions between TAMs and pericytes promote glioblastoma progression. (**A**) Pro-tumoral macrophages (M2 TAMs) induce upregulation of PDGFR-β in glioma cells, generating a PDGF-BB signaling loop that enhances pericyte recruitment. (**B**) Activation of the PDGF-BB/SOX7 axis in pericytes stimulates the release of IL-33. IL-33 induces polarization of TAMs to an immunosuppressive M2 phenotype and supports tumor angiogenesis. (**C**) Pericytes secrete other anti-inflammatory cytokines, such as IL-10 and TGF-β, that reinforce immune evasion. (**D**) CECR1 secreted by macrophages supports M2 TAM polarization and enhances PDGF-BB/PDGFR-β signaling. (*E*) The MCAM/CD163 axis mediates interactions between pericytes and TAMs, promoting macrophage reprograming toward a pro-tumoral phenotype. These reciprocal interactions foster immune suppression, angiogenesis, and glioblastoma progression. Abbreviations: TAMs, tumor-associated macrophages; GB, glioblastoma.

Pericytes also influence the polarization of TAMs. Yang et al. identified the PDGF-BB/SOX7/IL-33 axis as a mechanism for that aim. PDGF-BB signaling induces the upregulation of SOX7 in pericytes, which leads to IL-33 expression. IL-33 acts as polarizing factor that recruits TAMs and promotes their differentiation into an M2-like, pro-tumoral phenotype [68]. Additionally, Zhu et al. (2017) demonstrated that TAMs expressing extracellular adenosine deaminase (CECR1) enhance pericyte migration. In that case, the PDGF-B/PDGFR-β pathway creates a feedback loop that supports tumor vascularization. Their experimental model showed that silencing CECR1 in TAMs reduced pericyte migration by approximately 40% in in vitro transwell assays [26]. This type of interaction underscores the cooperative role of TAMs and pericytes in tumor progression (Figure 4).

The physical co-localization of TAM and pericyte markers within GB further supports their functional interplay. Immunohistochemical studies showed the co-expression of macrophage markers (CD163, CD68) and pericyte markers (NG2, MCAM) in perivascular regions of the tumor [9,29]. This spatial proximity suggests potential juxtracrine interactions. The MCAM/CD163 axis has been implicated in TAM polarization; silencing MCAM expression in pericytes increased the polarization of microglia toward an M1-like phenotype [9]. These findings indicate that pericyte-derived signals contribute to maintaining the immunosuppressive M2 state of TAMs.

Targeting pericyte–TAM interactions in preclinical studies showed some promising data. Scholz et al. (2016) demonstrated that a bispecific Ang-2/VEGF antibody shifted TAMs toward an anti-tumoral M1 phenotype in murine GB models. They found a reduction both in angiogenesis and in tumor progression. These findings highlight the therapeutic potential of modulating pericyte-mediated TAM polarization to combat the immunosuppressive tumor microenvironment of GB [23].

## 5. Therapeutic Implications Based on Pericyte-Driven Mechanisms

Pericytes have emerged as key players in the progression of GB. Their involvement extends beyond providing structural and functional support to tumor vasculature. Pericytes also maintain the BBB [41], take part in angiogenesis [31], and play a critical role in DNA damage repair mechanisms (DDR) [33,34]. Thus, it becomes evident that pericytes actively contribute to the tumor’s resistance to radiotherapy and chemotherapy and the short survival after surgery. Experimental studies using murine GB models have demonstrated that the genetic depletion of proliferating pericytes using ganciclovir significantly prolongs survival [16,33,69,70]. Although this concrete strategy failed during clinical trials, addressing therapeutic efforts to these peculiar cells represents an opportunity to weaken GB through increasing its sensibility to additional drugs. Briefly, some strategies are now highlighted as promising areas for pericyte research, along with potential challenges in translating these findings into clinical therapies (Table 1).

### 5.1. Targeting the Pericyte–Vascular Role

Pericytes play a crucial role in GB vascular co-option, a process by which tumor cells hijack pre-existing blood vessels to support their growth, bypassing the need for new blood vessel formation. GB co-option is particularly important after antiangiogenic therapies, as it enables tumors to evade treatment and contributes to recurrence as has been above discussed [51]. Recent findings highlight that direct interactions between glioblastoma cells and pericytes through filopodia-like structures are essential for vascular co-option. These interactions alter pericyte contractility and transcriptional profiles, promoting vascular remodeling and an immunosuppressive tumor microenvironment [12].

Improving BBB stability may complicate tumor vascular co-option. One strategy for that aim is the experimental activation of the Wnt-β-catenin signaling pathway through Gpr124 (G-protein-coupled receptor 124), a receptor crucial for cerebrovascular development. Gpr124 is an orphan G-protein-coupled receptor specifically expressed in endothelial cells of the CNS vasculature. It is a regulator of the integrity and maintenance of the BBB by modulating endothelial–pericyte interactions. Activation of Gpr124 has been shown to reduce the risk of microvascular hemorrhage and restore endothelial tight junctions and pericyte coverage [71]. Similarly, the extracellular matrix protein EGFL7 (epidermal growth factor-like protein 7), secreted by blood vessels in GB, has been shown to enhance pericyte coverage and stabilize tumor vasculature, reducing vascular permeability [72]. These works suggest interest in therapies aimed at enhancing the stability and function of tumor vasculature in GB in order to improve the effectiveness of antiangiogenic agents.

Another compelling target is BMX, a tyrosine kinase highly expressed in pericytes derived from GSCs. BMX regulates self-renewal and tumorigenesis. Its inhibition with ibrutinib increases drug delivery into tumors and also enhances the effects of Bevacizumab in GB xenograft models [15,73]. These findings suggest that targeting BMX, particularly in pericytes derived from GSCs, could offer a highly selective approach, improving the efficacy of chemotherapy by facilitating the better penetration of drugs through the blood–brain barrier (BBB). Additionally, BMX inhibition represents a potential way to specifically target neoplastic pericytes without affecting the integrity of normal brain vasculature [74].

### 5.2. Targeting DNA Damage Repair Through the CCL5-CCR5 Axis

The CCL5-CCR5 signaling reinforces the DNA damage repair (DDR) mechanisms in GB promoting tumor cell survival and resistance to chemotherapy. The chemokine CCL5 is secreted by cells in the tumor microenvironment (TME), including pericytes. CCL5 binds to CCR5 on GB cells enhancing tumor cell migration and the activation of the non-homologous end joining (NHEJ) DDR pathway, as aforementioned [33]. Disrupting the CCL5-CCR5 axis represents a promising therapeutic strategy. This pathway could be disrupted using various interfering approaches. Maraviroc, a drug approved for HIV treatment, acts as a CCR5 antagonist preventing CCR5 initialization of DDR signaling in GB cells [33,34]. This strategy showed promising preclinical results [35], but its efficacy was not confirmed in clinical trials. Although maraviroc is not currently been used, the same strategy is under study with different drug combinations [34]. It is possible to inhibit CCR5 in GB cells, or to interfere with the CCL5 secreted by pericytes. CCL5/CCR5 inhibitors prevent the downstream phosphorylation of AKT and the activation of DNA-PKcs, disrupting the NHEJ pathway in GB cells. This interference may enhance the cytotoxic effects in tumor cell death with current therapies like TMZ and/or radiation [35]. Targeting this axis may weaken GB resistance mechanisms by reducing the tumor’s capacity to survive under therapeutic stress.

### 5.3. Hijacking Pericytes to Modulate the Immune System

The potential to “hijack” pericytes and reprogram them to modulate the immune system represents a promising therapeutic strategy in cancer. This approach, though still in its theoretical stages, holds intriguing potential. Therefore, it seems of interest to introduce it within the context of this literature review. Pericytes are highly migratory, and they are involved in processes such as vascular stabilization and immune regulation. They are also highly plastic and can adopt either pro- or anti-tumor roles, depending on the specific signals mediated by the tumor microenvironment. These facts suggest that their reprograming could potentially create a more pro-inflammatory or immunosupportive microenvironment against tumor growth [75]. For instance, signaling molecules such as PDGF-β (platelet-derived growth factor beta) and TNFSF14 (tumor necrosis factor superfamily member 14, also known as LIGHT) have been implicated in shifting pericytes from an angiogenic and proliferative state to a more mature, contractile form [76]. This shift is needed for vascular normalization, offering another potential therapeutic avenue [76]. A particularly interesting mechanism for reprograming pericytes involves targeting Cdc42 (cell division control protein 42). Cdc42 is a small GTPase that regulates cell polarity, migration, and cytoskeletal dynamics. Silencing Cdc42 with siRNA has been shown in vitro to reprogram pericytes, changing their phenotype from a tumor-promoting, angiogenic state to a tumor-suppressive one, resembling pro-inflammatory M1 macrophages. This shift enhances the innate immune response against a tumor and prevents vascular co-option [49,51] and, altogether, highlights that the potential of modulating the function of pericytes can transform their role from a tumor-supportive to a tumor-suppressive phenotype.

Recent advancements have also identified tumor-specific variants of NG2 (neural/glial antigen 2 or chondroitin sulfate proteoglycan 4). These NG2 variants appear to be selectively expressed in tumor-associated pericytes but are absent in normal brain vasculature. This fact makes them attractive targets for selective therapies. Targeting these variants could potentially disrupt pericyte–tumor interactions, reduce angiogenesis, and shift the immune balance in the tumor microenvironment [27,29].

While these hijacking strategies are still in the early stages of investigation and not widely studied in glioblastoma, they represent an exciting unexplored area of research. Reprograming pericytes to adopt an anti-tumor immune phenotype or to normalize the tumor vasculature could provide novel therapeutic approaches, enhancing the effectiveness of existing treatments and potentially reducing tumor progression.

## 6. Future Directions

The complexity of the glioblastoma TME and the multifaceted roles of pericytes offer both opportunities and challenges for future research. Current therapeutic strategies aim to target pericyte-driven mechanisms. However, further investigation is essential, especially to address the following areas:

*Understanding Pericyte Heterogeneity:* Pericytes within GB are not a homogeneous population. Identifying specific subtypes, such as type-1 (angiogenesis-supportive) and type-2 (proangiogenic) ones, could improve therapeutic precision [31,54]. Single-cell RNA sequencing (scRNA-seq) can clarify pericyte heterogeneity and its role in tumor progression. Advances in tumor-specific biomarkers, such as NG2 variants or GT198+ pericytes, may offer new opportunities for selective targeting [48,77]. Interestingly, a recent study on neonatal brains shows that pericytes respond to hypoxia, promoting vascular repair and tissue regeneration [78]. This highlights the interest in how these mechanisms may influence pediatric gliomas.

*Targeting Pericyte Plasticity:* Pericytes demonstrate remarkable plasticity within the hypoxic and immunosuppressive TME. Under those circumstances, they may potentially acquire fibroblast-like or macrophage-like phenotypes [75,79]. A recent study showed that GB-secreted TFG-b induces metabolic reprograming in pericytes [41], contributing to a chaotic angiogenesis and the disruption of the BBB [80]. Modulating both its own molecular pathways or the exosome vesicles secreted may reduce their reprograming capacities.

*Pericyte–Immune System Interactions*: Pericytes contribute to GB immunosuppression by inhibiting T-cell activation and promoting macrophage polarization toward an M2-like phenotype. Targeting pathways such as the MCAM-CD163 axis or PDGF-BB/SOX7/IL-33 could reverse this immunosuppression, enhance T-cell infiltration, and disrupt tumor vascular support. Strategies based on the metabolic reprograming of pericytes may also improve immunotherapy efficacy [9,75].

The investigation of pericytes as therapeutic targets in glioblastoma is still in its early stages. Translating pericyte-targeted therapies from preclinical models to clinical practice remains challenging. Advances in single-cell technologies, molecular profiling, and targeted therapies hold promise for overcoming the current limitations. Addressing these challenges could pave the way for innovative treatment strategies that improve patient outcomes in this devastating disease.

## 7. Conclusions

This review highlights the clinical potential of targeting pericytes, not only by disrupting angiogenesis but also by inhibiting the vascular co-option process or the DDR response, critical for GB progression. The emerging evidence surrounding the multifactorial role of pericytes in GB suggests that they are key players in tumor progression, angiogenesis, and therapeutic resistance, with an interesting role in immune modulation. Combining pericyte-targeted strategies with established therapies such as antiangiogenics and TMZ and immune checkpoint inhibitors may improve treatment efficacy and delay the inevitable recurrence. Further studies are needed to explore these mechanisms and their translational potential in glioblastoma. With advances in understanding the specific molecular signatures of tumor-associated pericytes, therapies that selectively target these cells are becoming increasingly viable, offering new hope for the treatment of this devastating disease.

## Figures and Tables

**Table 1:** Summary of therapeutic strategies.

Mechanism	Therapeutic Strategy	Target	Challenges and Considerations
***Pericyte–vascular role***			
Vascular co-option	Wnt-β-catenin activation (Gpr124 modulation)	Endothelial–pericyte signaling	Restoring BBB integrity without impairing CNS vasculature
Angiogenesis	VEGF blockade + multi-kinase inhibitors *	VEGF, PDGF, FGF pathways	Limited efficacy in pediatric gliomas, adverse effects
Pericyte–tumor interaction	BMX kinase inhibition	BMX kinase in GSC-derived pericytes	Selective targeting of tumor-associated pericytes
***DNA damage repair mechanism***			
DDR mediated by CCL5-CCR5 axis	CCL5 secretion inhibition or CCR5 blockade	CCL5-secreting pericytes or CCR5-expressing GB cells	Off-target effects, resistance in advanced tumors
***Modulation of the immune system***			
Pericyte reprograming	Cdc42 siRNA silencing	Pericyte phenotype switching	Translational barriers for immune-targeted therapies
Pericyte reprograming	TNFSF14 activation	Tumor-associated pericytes	Potential off-target effects, limited preclinical validation in glioblastoma

* All listed therapeutic strategies are currently in preclinical stages, with the exception of VEGF blockade and multi-kinase inhibitors.
